# Impact of Cardiac Magnetic Resonance on the Diagnosis of Left Ventricular Noncompaction—A 15-Year Experience

**DOI:** 10.3390/jcm13040949

**Published:** 2024-02-07

**Authors:** Natalia Ojrzyńska-Witek, Magdalena Marczak, Łukasz Mazurkiewicz, Joanna Petryka-Mazurkiewicz, Barbara Miłosz, Jacek Grzybowski, Mateusz Śpiewak

**Affiliations:** 1 Department of Cardiomyopathy, National Institute of Cardiology, 04-628 Warsaw, Poland; lmazurkiewicz@ikard.pl (Ł.M.); jgrzybowski@ikard.pl (J.G.); 2 Magnetic Resonance Unit, National Institute of Cardiology, 04-628 Warsaw, Poland; mmarczak@ikard.pl (M.M.); jpetryka@ikard.pl (J.P.-M.); bmilosz@ikard.pl (B.M.); mspiewak@ikard.pl (M.Ś.)

**Keywords:** left ventricular noncompaction, cardiac magnetic resonance, late gadolinium enhancement, cardiomyopathy

## Abstract

The aim of this study was to assess the impact of cardiac magnetic resonance (CMR) on the diagnosis in patients with known or suspected left ventricular noncompaction (LVNC). We retrospectively reviewed the medical charts of 12,811 consecutive patients who had CMR studies between 2008 and 2022 in a large tertiary center. We included patients referred for CMR because of known or suspected LVNC. The study sample consisted of 333 patients, 193 (58.0%) male, median age 39.0 (26.8–51.0) years. Among 74 patients fulfilling the echocardiographic LVNC criteria, the diagnosis was confirmed in 54 (73.0%) cases. In 259 patients with ultrasound-based suspicion of LVNC, CMR led to an LVNC diagnosis in 82 (31.7%) patients. In both groups, CMR led to a new diagnosis in 89 cases (10 (13.5%) and 79 (30.5%)). A quantity of 38 (5.4%) patients were diagnosed with dilated cardiomyopathy, 11 (1.4%) patients were diagnosed with hypertrophic cardiomyopathy, and 21 (4.1%) patients were diagnosed with unclassified cardiomyopathy. In four patients with suspected LVNC, a myocardial trabeculation was a secondary result of dilatation due to coronary heart disease. In five cases, valvular heart disease was found. Four patients were diagnosed with athlete’s heart. Other diagnoses (arrhythmogenic right ventricular cardiomyopathy, peripartum cardiomyopathy, hypokinetic non-dilated cardiomyopathy, sarcoidosis, amyloidosis, and ventricular septum defect) were found in six patients. CMR is a valuable tool in the evaluation of cardiac muscle and in differentiating LVNC and other cardiac diseases.

## 1. Introduction

Cardiomyopathies encompass a heterogeneous group of diseases affecting the cardiac muscle. One of them is left ventricular noncompaction (LVNC), wherein the myocardium has a bilayered structure with a compacted thin epicardial layer and a much thicker, trabeculated noncompacted endocardial layer with deep recesses [1]. LVNC can be isolated or can coexist with congenital heart disease [2].

LVNC is a rare disease, and its prevalence remains unknown but is higher than expected; it varies between 1.28% according to echocardiography and 14.79% according to CMR [3]. 

LVNC is associated with a risk of arrhythmias, thromboembolic events, and heart failure [4,5]. The risk of adverse events is associated with systolic dysfunction, not the extent of the trabeculated myocardium, and is similar for patients with LVNC and dilated cardiomyopathy (DCM) [6,7,8]. The clinical outcome is also associated with the presence of myocardial scarring [7,9]. Implantable cardioverter-defibrillator (ICD) implantation for the primary prevention of sudden cardiac death (SCD) in patients with LVNC should be in line with the recommendation for DCM and hypokinetic nondilated cardiomyopathy [10].

According to the ESC guidelines [11], cardiac magnetic resonance (CMR), due to its ability to visualize myocardial tissue, is recommended, inter alia, in patients with LVNC.

This study aimed to assess the impact of CMR on the diagnosis in patients with known or suspected LVNC according to previously performed echocardiography. We sought to assess the impact of confirmed LVNC or a new diagnosis on clinical management.

## 2. Materials and Methods

### 2.1. Study Design

We retrospectively reviewed all the medical charts of consecutive patients who underwent CMR between 2008 and 2022 at a large tertiary center (National Institute of Cardiology, Warsaw, Poland). We included all patients referred for CMR because of known or suspected LVNC based on a previous echocardiogram.

The echocardiographic criterion used for diagnosing LVNC was a noncompacted/compacted (NC/C) layer ratio >2 in the myocardium with a two-layer structure [12]. LVNC was diagnosed in patients who fulfilled those criteria. In some cases, patients with suspected LVNC (poor acoustic window, borderline NC/C ratio, etc.) were referred for further evaluation via CMR.

CMR images were reviewed, and the final diagnoses were made using all available data.

### 2.2. Ethics Approval

All patients provided written informed consent for the CMR study.

The study was approved by the Bioethics Committee of the National Institute of Cardiology, decision number IK.NPIA.0021.25.2024/23.

### 2.3. CMR Protocol

All CMR exams were performed on a 1.5 T scanner (Avanto/Avanto^fit^, Siemens, Erlangen, Germany). A gadolinium-based contrast agent at the standard dosage (0.1 mmol kg) was given intravenously to all patients who had no contraindications.

Long-axis and short-axis electrocardiogram-gated breath-hold cine images were taken for chamber volumetric and functional assessments.

LVNC was diagnosed using criteria proposed by Petersen et al. [13] in patients in whom the noncompacted (NC)/compacted (C) end-diastolic layer measurement ratio was greater than or equal to 2.3 [13].

All measurements and all analyses were performed by experienced physicians.

### 2.4. Statistical Analysis

Statistical analyses were performed using MedCalc 19.4.1 (MedCalc Software Ltd., Mariakerke, Belgium). The normality of the distribution of continuous variables was checked using the Kolmogorov–Smirnov test. Nonnormally distributed continuous data were tested with the Mann–Whitney test and were presented as the median (interquartile range [IQR]). The Kruskal–Wallis test with the post hoc Conover test was used to compare independent groups. The chi-square test was used to compare categorical variables.

A two-tailed *p* value < 0.05 was considered statistically significant.

## 3. Results

### 3.1. Baseline Characteristics

Out of 12811 CMR exams of consecutive patients who underwent CMR between 2008 and 2022 in our hospital, 333 (2.6%) patients met the inclusion criteria, 193 (58.0%) of whom were male. The median age was 39.0 (26.8–51.0) years.

All patients were Caucasian, and 99.1% were of Polish origin.

According to the echocardiography results, LVNC was suspected in 259 patients and confirmed in 74 patients.

The patients with known vs. suspected LVNC differed significantly in age, left ventricular end-systolic volume (LVESV) and left ventricular ejection fraction (LVEF). There were no difference in the presence of late gadolinium enhancement (LGE), left ventricular mass (LVM), left ventricular end-diastolic volume (LVEDV), or measured right ventricular volumes. 

The baseline characteristics are presented in Table 1.

### 3.2. CMR Diagnosis

LVNC was confirmed in 136 (40.8%) patients: 82 (31.7%) with suspected (based on echocardiography) and 54 (73.0%) with previously diagnosed LVNC.

**Figure 1:**
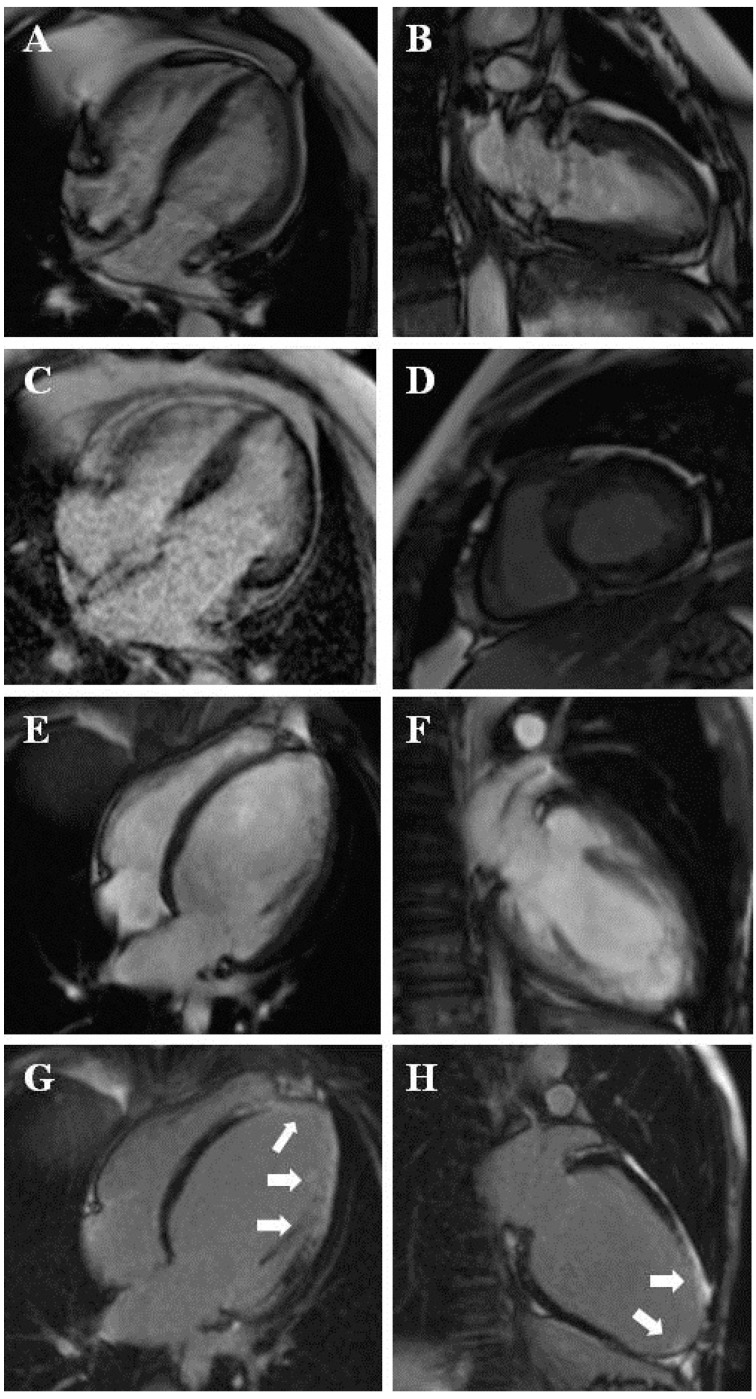
(**A**–**D**) Hypertrophic cardiomyopathy, with LGE in hypertrophied segments (**C**,**D**). (**E**–**H**) Coronary heart disease with a secondary myocardial trabeculation, with subendocaridal to transmural LGE in basal inferolateral, mid-inferolateral, mid-anterolateral, apical segments, and the apex (arrows) (**G**,**H**).

**Figure 2:**
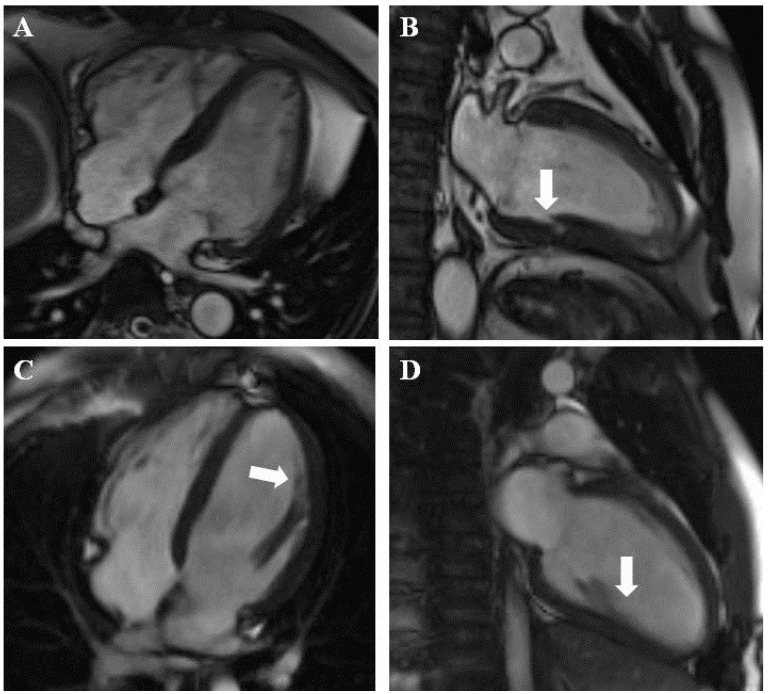
(**A**,**B**) Other, nonspecific cardiomyopathy: abnormal left ventricular muscle structure and myocardial crypt (arrow) in the posterior wall. (**C**,**D**) Example of normal CMR study. The broad base of the anterolateral papillary muscle (arrows) may mimic hypertrabeculation.

In 89 patients, CMR led to a new diagnosis. DCM was diagnosed in 38 patients: 34 (13.1%) with ultrasound-based suspicion of LVNC and 4 (5.4%) with known (according to echocardiography) LVNC. Eleven patients in these two groups were diagnosed with hypertrophic cardiomyopathy (HCM) (10 (3.9%) and 1 (1.4%)) (Figure 1A–D), and 21 were diagnosed with unclassified cardiomyopathy (18 (6.9%) and 3 (4.1%)) (Figure 2A,B).

In four (1.5%) patients with suspected LVNC, myocardial trabeculation was a secondary result of dilatation due to coronary heart disease (Figure 1E–H).

In five patients of these two groups, valvular heart disease (four (1.5%) and one (1.4%)) was found. Four patients (three (1.2%) and one (1.4%)) were diagnosed with athlete’s heart.

Other diagnoses (arrhythmogenic right ventricular cardiomyopathy, peripartum cardiomyopathy, hypokinetic nondilated cardiomyopathy, sarcoidosis, amyloidosis, and ventricular septum defect) were made in six patients with echocardiographic suspicion of LVNC.

In 108 patients (suspected group, 98 (37.8%); confirmed group, 10 (13.5%)) the CMR scan was normal or nonspecific (Figure 2C,D).

**Figure 3:**
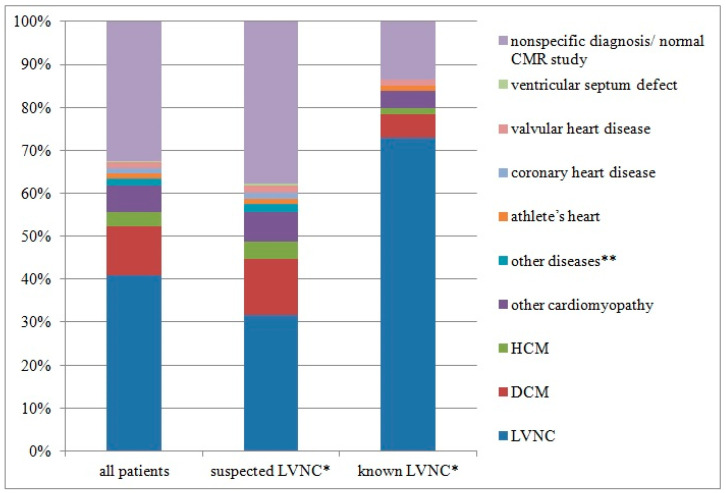
Final diagnoses. * Based on echocardiography. ** Arrhythmogenic right ventricular cardiomyopathy, peripartum cardiomyopathy, hypokinetic non-dilated cardiomyopathy, sarcoidosis, amyloidosis, ventricular septum defect. CMR, cardiac magnetic resonance; DCM, dilated cardiomyopathy; HCM, hypertrophic cardiomyopathy; LVNC, left ventricular non-compaction.

All diagnoses are presented in Figure 3.

### 3.3. Patients with Confirmed and Unconfirmed LVNC Diagnoses

We compared groups with suspected and known LVNC that was confirmed or not confirmed by CMR (Table 2). There were statistically significant differences between the groups in LVESV (*p* = 0.003) and LVEF (*p* = 0.001), when comparing patients with LVNC diagnosed previously on echocardiography but unconfirmed in CMR with patients with echocardiographic suspicion of LVNC or an echocardiographic-based diagnosis of LVNC, both of which were confirmed in CMR. There was no significant difference in age in comparison with patients with known vs. suspected LVNC according to echocardiography.

When analyzing differences between all patients with and without LVNC, differences were observed in both end-systolic and end-diastolic LV volumes and LV ejection fractions (Table 3).

### 3.4. Patients with Dilated Cardiomyopathy, Hypertrophic Cardiomyopathy, and Left Ventricular Noncompaction

According to the ESC guidelines for the diagnosis and treatment of heart failure [11], LVNC is diagnosed in patients with a family history of DCM/HCM, and phenotypes of those cardiomyopathies overlap. Thus, we performed statistical analyses among these groups (Table 4).

There were statistically significant differences between the groups in terms of left and right ventricular volumes, ejection fractions, and age. There were no difference in the presence of LGE.

**Figure 4:**
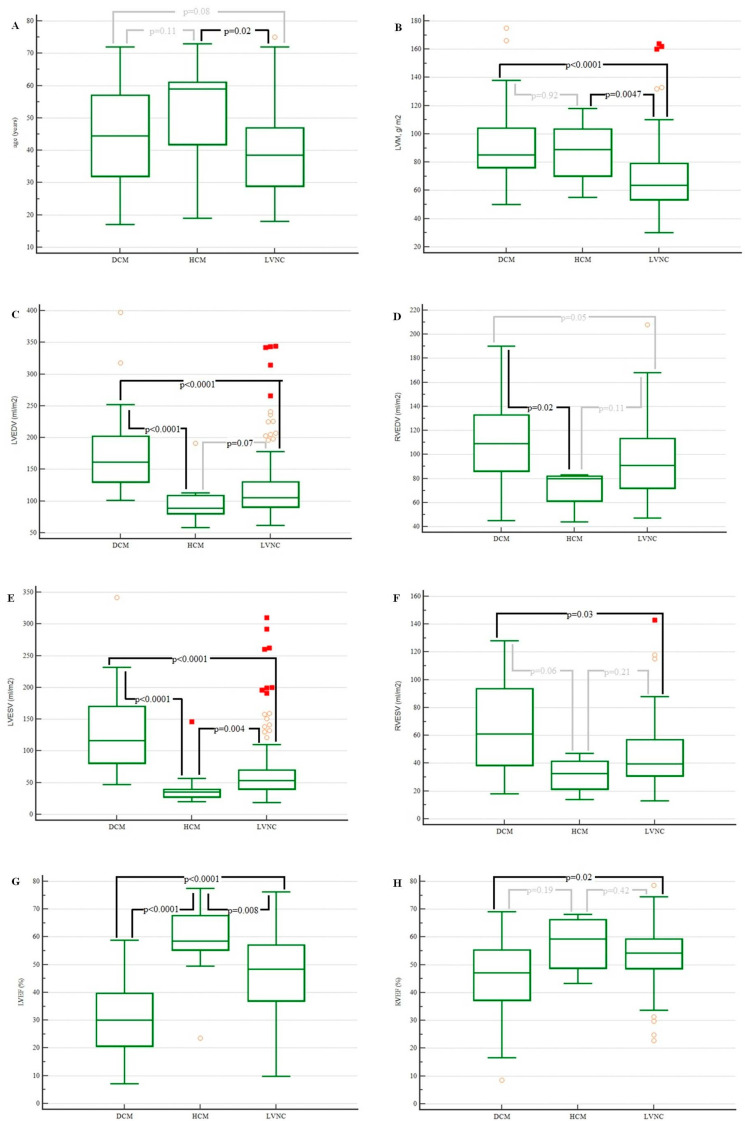
Age, left ventricular end-diastolic and left ventricular end-systolic volumes, left ventricular ejection fraction, left ventricular mass, right ventricular end-diastolic and right ventricular end-systolic volumes, and right ventricular ejection fraction of patients with LVNC, HCM, and DCM. Box plots with median and IQR, where whiskers are the minimum and the maximum and dots represent outliers. DCM, dilated cardiomyopathy; HCM, hypertrophic cardiomyopathy; LGE, late gadolinium enhancement; LVEDV, left ventricular end-diastolic volume; LVEF, left ventricular ejection fraction; LVEDV, left ventricular end-diastolic volume; LVNC, left ventricular noncompaction; LVNC, left ventricular non-compaction; LVM, left ventricular mass; RVEDV, right ventricular end-diastolic volume; RVEF, right ventricular ejection fraction; RVEDV, left ventricular end-diastolic volume. (**A**). Age of patients with DCM, HCM and LVNC; (**B**). LWM of patients with DCM, HCM and LVNC; (**C**). LVEDV of patients with DCM, HCM and LVNC; (**D**). RVEDV of patients with DCM, HCM and LVNC; (**E**). LVESV of patients with DCM, HCM and LVNC; (**F**). RVESV of patients with DCM, HCM and LVNC; (**G**). LVEF of patients with DCM, HCM and LVNC; (**H**). RVEF of patients with DCM, HCM and LVNC.

Patients diagnosed with LVNC were significantly younger (Figure 4A) than patients diagnosed with HCM were.

The left ventricular mass was lower in patients with LVNC compared with the patients with DCM and HCM (Figure 4B).

Left ventricular end-diastolic volume and right ventricular end-diastolic volume were lower in patients with LVNC and with DCM (Figure 4C,E,F). There were statistically significant differences in the left ventricular end-systolic volume (*p* value < 0.0001; Figure 4G) and right ventricular ejection fraction (*p* value = 0.02; Figure 4H) between those groups of patients.

## 4. Discussion

Although the first LVNC case was described almost a century ago, there is ongoing discussion about its classification and diagnostic criteria [1]. The WHO described LVNC as unclassified cardiomyopathy [14], while the American Heart Association classified LVNC as a primary genetic disorder [15], and according to the European Society of Cardiology (ESC) working group, LVNC belongs to the unclassified cardiomyopathies [16]. There are also doubts concerning nomenclature. Recently, cardiovascular imaging experts suggested using the term “excessive trabeculation” or “hypertrabeculation” instead of LVNC [17,18].

Since LVNC occurs in families with DCM and HCM phenotypes, the ESC guidelines suggest that LVNC is a rare subtype of DCM/HCM [11] and that the presence of noncompacted myocardium does not change management or patient prognosis [17].

In our cohort, DCM and HCM were found in 49 patients (14.7%). CMR-based measurements (volumes, ejection fractions, and masses) differed significantly between those groups (Table 4 and Figure 4), especially when comparing patients with LVNC and DCM.

Given the lack of a diagnostic gold standard, differentiating between LVNC and excessive but normal trabeculation poses a challenge. The CMR criterion used for diagnosing LVNC was a noncompacted/compacted layer ratio ≥2.3 [13], and the echocardiographic criterion was an NC/C ratio >2 [12]. This could be one of the reasons why a smaller group of patients fulfilled the CMR criteria for an LVNC diagnosis than fulfilled the echocardiographic LVNC criteria.

These results differ from those from the meta-analysis of LVNC prevalence, which suggested the overdiagnosis of LVNC while using CMR and the underdiagnosis on echocardiography [3].

CMR provided better insight into the myocardial structure than echocardiography and led to the differentiation of myocardial hypertrabeculation and other structures (Figure 2). CMR also had the ability to characterize the myocardial structure and the presence, type, and extent of fibrosis. LGE imaging was crucial in the diagnosis of coronary heart disease and amyloidosis and was helpful in the diagnosis of other diseases, such as HCM (Figure 1).

Furthermore, changes in the diagnosis led to clinical implications.

A new CMR-based HCM diagnosis was associated with a need for further clinical management (evaluating SCD risk, indicating ICD implantation indication, family screening, etc.) [19].

The recommendation of an ICD, such as for LVEF ≤ 35%, for DCM should also be applied in patients with LVNC [10]. The presence of LGE in patients is associated with a higher risk of ventricular arrhythmias and other adverse events [20,21].

In our group of patients with CMR-confirmed LVNC, 29 patients had LVEF ≤ 35%. LGE was seen in 75% of them (21 of 28 patients in whom the gadolinium-based contrast agent was administered). In the DCM group, LVEF ≤ 35% was seen in 21 patients. Ninety percent of them (18/20 patients who underwent CMR with contrast) had an observed area of LGE.

Myocardium hypertrabeculation can be seen quite often in athletic individuals as a cardiac adaptation to increased preload [22], so the diagnostic criteria for athletes are different [23]. In our cohort, four patients were diagnosed with athlete’s heart. In those patients, the LVNC diagnosis would prompt annual follow-up, risk stratification, and in some cases, restriction of sport activities [23].

In our cohort, CMR led to a new diagnosis in 89 (26.7%) patients. In the EuroCMR registry, Bruder et al. reviewed more than 27,000 consecutive patients who underwent CMR [24]. The final diagnosis differed from the pre-CMR diagnosis in 8.7% of patients [24]. In another study evaluating the impact of CMR in patients with heart failure of unknown etiology, the CMR-based diagnosis was different from the pre-CMR diagnosis in 38.7% of patients [25].

In our study, CMR imaging impacted 59.2% of the patients (a new diagnosis and normal CMR study/nonspecific CMR findings). According to the Euro-CMR registry, CMR impacted patient management in 61.8% of patients [24].

### Study Limitations and Strengths

This study has several limitations inherent to retrospective studies. We collected data from only one hospital; therefore, our cohort was limited by one site and one race and did not represent all LVNC patients.

We also had no follow-up data.

The diagnostic criteria for LVNC are still under debate. We used the Petersen criteria [13], while the diagnostic criteria proposed by Jacquier et al. for the diagnosis of LVNC were based on measurements of mass (trabeculated LVM above 20% of the global LVM) [23]. The chosen methods impacted the results.

To our knowledge, this is the first study assessing the clinical utility of CMR in Polish patients with suspected or previously diagnosed left ventricular noncompaction based on echocardiography.

## 5. Conclusions

Our study suggests that CMR had a true impact on the diagnosis in both groups with an echocardiography-based diagnosis and suspicion of LVNC. The LVNC diagnosis was confirmed in 40.8% of the patients. The CMR study impacted 59.2% of patients: in 26.7% of cases it led to a new diagnosis, and in 32.4% of cases it was normal or nonspecific.

Our study highlights the importance of CMR in the assessment of LVNC patients.

## Figures and Tables

**Table 1:** Patients’ characteristics. LGE, late gadolinium enhancement; LVEDV, left ventricular end-diastolic volume; LVEF, left ventricular ejection fraction; LVEDV, left ventricular end-diastolic volume; LVNC, left ventricular non-compaction; LVM, left ventricular mass; RVEDV, right ventricular end-diastolic volume; RVEF, right ventricular ejection fraction; RVEDV, left ventricular end-diastolic volume. ^a^ The results are presented as the median (interquartile range [IQR]). ^b^ There were 14 patients (4.2%) in whom the gadolinium-based contrast agent was not administered.

Variable	All Patients (n = 333)	Suspected LVNC (n = 259)	Known LVNC (n = 74)	*p* Value (Suspected vs. Known LVNC)
Male sex, n (%)	193 (58.0)	149 (57.5)	44 (59.5)	0.88
Age, years ^a^	39.0 (26.8–51.0)	39.0 (27.0–51.8)	34.5 (23.0–43.0)	0.03
LVEDV, mL/m^2 a^	102.0 (85.0–127.3)	99.0 (83.0–128.0)	108.0 (93.0–126.0)	0.05
LVESV, mL/m^2 a^	47.0 (35.0–68.5)	44.5 (34.0–67.0)	54.0 (41.0–73.0)	0.008
LVEF, % ^a^	54.1 (41.1–60.3)	55.5 (42.4–61.0)	49.8 (36.1–57.4)	0.0049
RVEDV, mL/m^2 a^	92.0 (77.0–113.0)	91.0 (77.0–113.0)	95.0 (72.8–109.5)	0.73
RVESV, mL/m^2 a^	41.0 (30.0–56.0)	42.0 (30.0–56.0)	40.0 (32.8–55.0)	0.83
RVEF, % ^a^	54.4 (48.6–60.3)	54.3 (48.5–61.2)	55.3 (48.9–59.4)	0.92
LGE, % (n) ^b^	44.8 (143/319)	46.0 (115/250)	40.6 (28/69)	0.88
LVM, g/ m^2 a^	64.5 (54.0–82.0)	66.0 (54.0–83.0)	60.0 (53.3–79.3)	0.19

**Table 2:** Comparison between groups with suspected and known LVNC confirmed and unconfirmed in CMR. LGE, late gadolinium enhancement; LVEDV, left ventricular end-diastolic volume; LVEF, left ventricular ejection fraction; LVEDV, left ventricular end-diastolic volume; LVNC, left ventricular non-compaction; LVM, left ventricular mass; RVEDV, right ventricular end-diastolic volume; RVEF, right ventricular ejection fraction; RVEDV, left ventricular end-diastolic volume. ^a^ The results are presented as the median (interquartile range [IQR]). ^b^ There were 14 patients (4.2%) in whom the gadolinium-based contrast agent was not administered.

Variable	Suspected LVNC Based on Echocardiography (n = 259)		Known LVNC Based on Echocardiography (n = 74)		*p* Value
	LVNC Confirmed in CMR (n = 82)	LVNC Unconfirmed in CMR (n = 177)	LVNC Confirmed in CMR (n = 54)	LVNC Unconfirmed in CMR (n = 20)	
Male sex, n (%)	43 (52.4)	106 (59.9)	34 (63.0)	10 (50.0)	0.89
Age, years ^a^	39.0 (32.0–51.0)	39.0 (24.0–52.0)	36.0 (27.0–43.0)	28.5 (19.5–50.0)	0.09
LVEDV, mL/m^2 a^	104.0 (87.0–130.0)	97.0 (83.0– 127.3)	108.0 (93.0–135.0)	106.0 (94.0–123.5)	0.12
LVESV, mL/m^2 a^	50.0 (38.0–67.3)	40.0 (33.0–67.0)	56.0 (42.0–81.0)	49.0 (36.0–62.5)	0.003
LVEF, % ^a^	50.0 (41.7–59.4)	56.7 (42.7–61.4)	46.7 (35.1–55.7)	54.0 (46.3–60.0)	0.001
RVEDV, mL/m^2 a^	86.0 (70.8–114.3)	92.0 (78.8–112.3)	93.0 (72.5–107.0)	104.0 (72.3–129.5)	0.91
RVESV, mL/m^2 a^	40.0 (30.0–59.0)	42.0 (29.0–56.0)	38.5 (32.5–54.0)	49.0 (33.5–58.5)	0.98
RVEF, % ^a^	53.8 (48.7–61.2)	54.8 (48.3–61.2)	55.7 (49.3–59.3)	53.5 (48.2–60.9)	0.98
LGE, % (n) ^b^	48.1 (38/79)	45.0 (77/171)	43.1 (22/51)	33.3 (6/18)	0.50
LVM, g/ m^2 a^	64.0 (53.0–81.0)	67.0 (55.0–83.0)	61.5 (55.0–77.0)	57.0 (47.5–82.3)	0.35

**Table 3:** Characteristics of patients divided according to the CMR-based final diagnosis. LGE, late gadolinium enhancement; LVEDV, left ventricular end-diastolic volume; LVEF, left ventricular ejection fraction; LVEDV, left ventricular end-diastolic volume; LVNC, left ventricular non-compaction; LVM, left ventricular mass; RVEDV, right ventricular end-diastolic volume; RVEF, right ventricular ejection fraction; RVEDV, left ventricular end-diastolic volume. ^a^ The results are presented as the median (interquartile range [IQR]). ^b^ There were 14 patients (4.2%) in whom the gadolinium-based contrast agent was not administered.

Variable	LVNC (n = 136)	Not LVNC (n = 197)	*p* Value
Male sex, n (%)	77 (56.6)	116 (58.9)	0.83
Age, years ^a^	38.5 (29.0–47.0)	39.0 (24.0–52.0)	0.63
LVEDV, mL/m^2 a^	105.5 (90.5–130.0)	97.0 (83.0–126.0)	0.047
LVESV, mL/m^2 a^	53.0 (40.0–70.0)	41.0 (33.0–67.0)	0.001
LVEF, % ^a^	48.4 (37.1–57.1)	56.5 (42.9–61.1)	0.0002
RVEDV, mL/m^2 a^	91.0 (72.0–113.3)	92.0 (78.0–113.0)	0.65
RVESV, mL/m^2 a^	39.5 (31.0–57.0)	42.0 (29.0–56.0)	0.83
RVEF, % ^a^	54.2 (48.7–59.3)	54.7 (48.4–61.0)	0.73
LGE, % (n) ^b^	46.2 (60/130)	43.9 (83/189)	0.81
LVM, g/ m^2 a^	63.5 (53.5–79.0)	66.0 (54.0–83.0)	0.38

**Table 4:** Comparison between groups with DCM, HCM, and LVNC. DCM, dilated cardiomyopathy; HCM, hypertrophic cardiomyopathy; LGE, late gadolinium enhancement; LVEDV, left ventricular end-diastolic volume; LVEF, left ventricular ejection fraction; LVEDV, left ventricular end-diastolic volume; LVNC, left ventricular noncompaction; LVNC, left ventricular non-compaction; LVM, left ventricular mass; RVEDV, right ventricular end-diastolic volume; RVEF, right ventricular ejection fraction; RVEDV, left ventricular end-diastolic volume. ^a^ The results are presented as the median (interquartile range [IQR]). ^b^ There were seven patients (3.8%) in whom the gadolinium-based contrast agent was not administered.

Variable	DCM (n = 38)	HCM (n = 11)	LVNC (n = 136)	*p* Value
Male sex, n (%)	24 (63.2)	6 (54.5)	77 (56.6)	0.93
Age, years ^a^	44.5 (32.0–57.0)	59.0 (41.8–61.0)	38.5 (29.0–47.0)	0.02
LVEDV, mL/m^2 a^	161.5 (130.0–202.0)	89.0 (80.5–109.0)	105.5 (90.5–130.0)	<0.000001
LVESV, mL/m^2 a^	116.0 (81.0–170.5)	35.0 (27.5–39.5)	53.0 (40.0–70.0)	<0.000001
LVEF, % ^a^	30.1 (20.7–39.7)	58.4 (55.4–67.6)	48.4 (37.0–57.1)	<0.000001
RVEDV, mL/m^2 a^	109.0 (86.3–132.8)	80.0 (61.5–82.0)	91.0 (72.0–113.3)	0.03
RVESV, mL/m^2 a^	61.0 (38.5–93.8)	32.5 (21.5–41.5)	39.5 (31.0–57.0)	0.04
RVEF, % ^a^	47.1 (37.3–55.4)	59.3 (48.9–66.2)	54.2 (48.7–59.3)	0.049
LGE, % (n) ^b^	29/37	10/11	59/130	0.09
LVM, g/ m^2 a^	85.0 (76.3–104.0)	89.0 (70.3–103.5)	63.5 (53.5–79.0)	0.000002

## Data Availability

The data presented in this study are available upon reasonable request from the corresponding author.
